# ORIFICE (Interventional Radiotherapy for Face Aesthetic Preservation) Study: Results of Interdisciplinary Assessment of Interstitial Interventional Radiotherapy (Brachytherapy) for Periorificial Face Cancer

**DOI:** 10.3390/jpm12071038

**Published:** 2022-06-24

**Authors:** Luca Tagliaferri, Ilaria Giarrizzo, Bruno Fionda, Mario Rigante, Monica Maria Pagliara, Calogero Casà, Claudio Parrilla, Valentina Lancellotta, Elisa Placidi, Alessandra Salvati, Gabriella Macchia, Stefano Gentileschi, Maria Antonietta Blasi, Alessio Giuseppe Morganti, Francesco Bussu, Ketty Peris, Gaetano Paludetti, Vincenzo Valentini

**Affiliations:** 1 U.O.C. Radioterapia Oncologica, Dipartimento di Diagnostica per Immagini, Radioterapia Oncologica ed Ematologia, Fondazione Policlinico Universitario A. Gemelli IRCCS, 00168 Rome, Italy; luca.tagliaferri@policlinicogemelli.it (L.T.); calogero.casa@guest.policlinicogemelli.it (C.C.); valentina.lancellotta@policlinicogemelli.it (V.L.); elisa.placidi@policlinicogemelli.it (E.P.); vincenzo.valentini@unicatt.it (V.V.); 2 Istituto di Radiologia, Università Cattolica del Sacro Cuore, 00168 Rome, Italy; ilaria.giarrizzo01@icatt.it (I.G.); alessandra.salvati@guest.policlinicogemelli.it (A.S.); 3 U.O.C. Otorinolaringoiatria, Dipartimento di Scienze dell’Invecchiamento, Neurologiche, Ortopediche e della Testa-Collo, Fondazione Policlinico Universitario A. Gemelli IRCCS, 00168 Rome, Italy; mario.rigante@policlinicogemelli.it (M.R.); claudio.parrilla@policlinicogemelli.it (C.P.); gaetano.paludetti@unicatt.it (G.P.); 4 U.O.C. Oncologia Oculare, Dipartimento di Scienze dell’Invecchiamento, Neurologiche, Ortopediche e della Testa-Collo, Fondazione Policlinico Universitario A. Gemelli IRCCS, 00168 Rome, Italy; monicamaria.pagliara@policlinicogemelli.it (M.M.P.); mariaantonietta.blasi@unicatt.it (M.A.B.); 5 Gemelli Molise Hospital, Università Cattolica del Sacro Cuore, 86100 Campobasso, Italy; gabriella.macchia@unicatt.it; 6 Dipartimento Scienze della Salute della Donna, del Bambino e di Sanità Pubblica, Unità di Chirurgia Plastica, Fondazione Policlinico Universitario A. Gemelli IRCCS, 00168 Rome, Italy; stefano.gentileschi@policlinicogemelli.it; 7 Dipartimento di Medicina e Chirurgia Traslazionale, Facoltà di Medicina e Chirurgia, Università Cattolica del Sacro Cuore, 00168 Rome, Italy; 8 Istituto di Oftalmologia, Università Cattolica del Sacro Cuore, 00168 Rome, Italy; 9 Radiation Oncology, IRCCS Azienda Ospedaliero-Universitaria di Bologna, 40126 Bologna, Italy; alessio.morganti2@unibo.it; 10 Radiation Oncology, DIMES, Alma Mater Studiorum University of Bologna, 40126 Bologna, Italy; 11 Otolaryngology Division, Azienda Ospedaliero Universitaria Sassari, 07100 Sassari, Italy; fbussu@uniss.it; 12 Department of Medicine, Surgery and Pharmacy, Sassari University, 07100 Sassari, Italy; 13 U.O.C. Dermatologia, Dipartimento di Scienze Mediche e Chirurgiche, Fondazione Policlinico Universitario A. Gemelli IRCCS, 00168 Rome, Italy; ketty.peris@unicatt.it; 14 Istituto di Dermatologia, Università Cattolica del Sacro Cuore, 00168 Rome, Italy; 15 Istituto di Otorinolaringoiatria, Università Cattolica del Sacro Cuore, 00168 Rome, Italy

**Keywords:** eyelid, nose vestibule, lip, brachytherapy, interventional radiotherapy

## Abstract

(1) Background: Periorificial face cancer (PFC), defined as both squamous cell carcinoma (SCC) and basal cell carcinoma (BCC) arising around the eyelids, the nose vestibule and the lips, has very high incidence rates worldwide. The aim of our retrospective analysis, focusing on local control (LC) and patients’ degree of satisfaction with the cosmetic outcome, is to present the results of a single institutional series of patients affected by PFC and treated by interventional radiotherapy (brachytherapy–IRT). (2) Methods: We retrospectively evaluated patients affected by PFC who were treated at our Interventional Oncology Center (IOC) with interstitial IRT from 2012 to 2021 with doses and volumes specific for each subsite considered. (3) Results: We report the results of 40 patients affected by PFC and treated by HDR interstitial IRT. The median follow-up was 24 months. The actuarial 3-year LC was 94%. Regarding patients’ satisfaction, we found that 93% of patients were satisfied and only 7% of patients were not completely satisfied with the final cosmetic result. (4) Conclusions: Interstitial HDR IRT could be an effective therapeutic option providing adequate disease control and preventing potentially disfiguring surgical approaches. More numerous and standardized studies are warranted to confirm the available evidence.

## 1. Introduction

Periorificial face cancer (PFC), defined as both squamous cell carcinoma (SCC) and basal cell carcinoma (BCC) arising around the eyelids, the nose vestibule and the lips, is definitively challenging regarding the choice of the best therapeutic approach [1]. In fact, the face is a very frequent area for both SCC and BCC development due to several factors, such as exposure to the sun and the multiplicity of anatomical sites and subsites that are present in the face [2].

The standard treatment for PFC is radical surgery, which may be performed through different technical approaches (curettage, excision and Mohs micrographic surgery) but with the same goal: to obtain adequate free margins and to prevent recurrence [3]. In the literature, suggested excisional margins for PFC range from 4.75 mm (for low-risk BCC) to 13.25 mm (for high-risk SCC). It is easily understandable that, in the face, such margins could be associated with potentially disfiguring consequences [4].

Traditionally, a conservative approach for skin cancer in the face was considered only in cases when surgery was refused by the patient or was not feasible due to poor clinical conditions [5]; on the contrary, a recent paradigm shift occurred where international guidelines explicitly dictated that conservative approaches should be considered when the post-surgical cosmetic outcome might not be satisfactory [6,7].

Several authors have proposed to subdivide the regions of the face into three main zones, low-risk, intermediate-risk and high-risk, which reflect the risk of recurrence after radical treatment [8]. Moreover, five different major criteria have been identified to consider the tumor “high-risk”, and these criteria include: tumor location (the so-called “H zone”), tumor size, clinical appearance, tumor recurrence or persistence and tumor histology [9].

Such classification is of paramount importance because the therapeutic strategy for radiation oncologists dramatically changes when dealing with non-high-risk versus high-risk zones [10]. In particular, for non-high-risk zones of the face, HDR contact radiotherapy could be considered as a valid therapeutic option. In fact, there are several experiences reported in the literature regarding the use of HDR contact radiotherapy in SCC and BCC of the face with high control rates and optimal aesthetic outcomes, including our earlier series [11].

On the contrary, when dealing with the “H zone”, which includes mainly tumors arising in the eyelids, in the nose vestibule and in the lips, it is necessary to pursue an interstitial approach with interventional radiotherapy (IRT–brachytherapy). In particular, intensity-modulated IRT is a modern development of the traditional IRT that allows the delivery of high radiation doses to the tumor while reducing the risk of serious adverse events, thus further improving the clinical outcomes [12].

IRT has faced a slow but inexorable decline during the past few years due to a lack of adequate training, education and personnel; however, IRT is rapidly gaining more and more attention within the oncological community because of its favorable dosimetric properties, which allow the delivery of high curative doses, sparing the normal surrounding tissue [13].

The most common indications for interstitial IRT of PFC (H zone face cancers) include nasal vestibule, lip/cheek and eyelid carcinomas. In fact, in these anatomical sites, IRT has proven to be effective, resulting in both improved “organ sparing” and “function sparing”.

The aim of our retrospective analysis, focusing on local control (LC) and patients’ degree of satisfaction with the cosmetic outcome, is to present the results of a single institutional series of patients affected by PFC and treated by IRT.

## 2. Materials and Methods

We retrospectively evaluated patients affected by PFC (primary nasal vestibule, lip and eyelid carcinomas) who were treated at our Interventional Oncology Center (IOC) with interstitial IRT from 2012 to 2021. Squamous cell carcinoma and basal cell carcinoma were included into the analysis. Patients with clinical evidence of positive nodes were included if a neck surgical approach was considered feasible.

Forty patients were identified through the electronic database within the frame of the SKIN-COBRA system, which allows us to retrospectively retrieve anonymized patients’ data, thus fully respecting the General Data Protection Regulations (GDPR) [14].

Overall, 40 patients (26 primary nasal vestibule, 10 lip cancer and 4 eyelid cancer) were recruited. However, for statistical purposes, patients lost to follow-up or with follow-up of less than 6 months were excluded from analysis.

All patients included in the present report were initially evaluated by a multidisciplinary tumor board, always including otorhinolaryngologists and ophthalmologists; in addition, dermatologists and plastic surgeons were involved according to the primary tumor site.

All patients underwent a complete physical examination together with a biopsy of the primary lesion for histology.

Head-and-neck computed tomography (CT) or magnetic resonance (MRI) was requested to complete the staging. Patients were asked to provide informed consent both for the procedure and for the treatment, as well as for the use of personal data for scientific purposes. 

The choice of IRT over a surgical approach was made after multidisciplinary evaluation considering function, cosmesis and patient preference in order to achieve optimal overall results.

Toxicities were assessed according to the Common Terminology Criteria for Adverse Events (CTCAE) version 4.0 [15].

Since, in the literature, there is a lack of consensus regarding a definitive assessment scale for patients undergoing interstitial IRT, we routinely asked patients to provide a subjective evaluation of their cosmetic outcome. A scale ranging from 1 to 4 was used. The scale consisted of satisfaction staging, with 1 meaning very dissatisfied, 2 dissatisfied, 3 satisfied and 4 very satisfied [16].

Patients’ characteristics are reported in Table 1.

The implant procedure included three different phases: pre-planning, implantation and treatment planning/delivery (Table 2).

In all cases, we used flexible 6 Fr tubes, placed inside the tumor volume with roughly 10 mm spacing, in accordance to the Paris system rules, in order to provide adequate coverage to the gross target volume (GTV). We used additional margins, ranging between 5 and 20 mm, according to the histology and the anatomical location to define the final clinical target volume (CTV).

Local fixation was usually obtained with buttons sutured to the skin. The number of catheters varied according to the CTV size. The entire procedure was performed under local or general anesthesia according to the single patients’ clinical conditions.

In all cases, the CT simulation was performed on the day after the plastic tubes’ implantation to allow the resolution of the swelling due to the interventional procedure. After the CT simulation, the CTV was contoured and the catheters were digitally reconstructed. The treatment plan was calculated using Oncentra Brachy (Elekta, Stockholm, Sweden) and treatment delivery was performed using a high-dose-rate (HDR) afterloader (Elekta MicroSelectron or Flexitron); the Groupe Européen de Curiethérapie and European SocieTy for Radiotherapy & Oncology (GEC-ESTRO) recommendations for skin and for head and neck brachytherapy were followed [17,18].

The treatment protocol was different according to the primary site, as follows: (i) nasal vestibule: the total prescribed dose was 44 Gy in 14 fractions, 3 Gy per fraction (except the first and last ones: 4 Gy), 2 fractions per day (b.i.d.); (ii) lip: the total prescribed dose was 45 Gy in 9 fractions, 5 Gy per fraction, 2 fractions per day (b.i.d.); (iii) eyelid: the total prescribed dose was 49 Gy in 14 fractions, 3.5 Gy per fraction, 2 fractions per day (b.i.d.). In the nasal vestibule and the eyelid, the treatment was delivered 5 days per week. A summary of the primary sites, doses and fractionation is presented in Table 3. Catheter removal after treatment completion was performed without any complication.

## 3. Results

We report the results of 40 patients affected by PFC and treated by HDR interstitial IRT.

Patients’ characteristics are reported in Table 1.

The mean age was 66.5 years (range: 39–94 years), with a slight male prevalence (60% males and 40% females).

The most commonly involved anatomical subsite was the nasal vestibule (65%), followed by the lips (25%) and eyelids (10%).

The most common histology was SCC, with 75% of cases, compared to BCC with 25%.

In the case of eyelid, all patients were T1; regarding the lip, 70% were T1 and 30% T2, whereas in the case of the nose vestibule, 38% were T1, 58% were T2 and 4% were T3. For eyelid and lip, the staging system was the TNM; for the nose vestibule, it was the Wang staging system.

All patients had clinically negative nodes except for two patients, both affected by nasal vestibule SCC, who were treated by HDR interstitial IRT on the primary site and subsequently neck dissection. 

The overall mean CTV was 12.7 cm, with large variability according to the subsite: 15.8 cm, 10.6 cm and 0.8 cm were the mean CTVs of the nasal vestibule, the lip and the eyelid, respectively. Noticeably, no interruptions were necessary due to acute toxicity and the scheduled fractions were correctly delivered to all patients.

**Figure 1:**
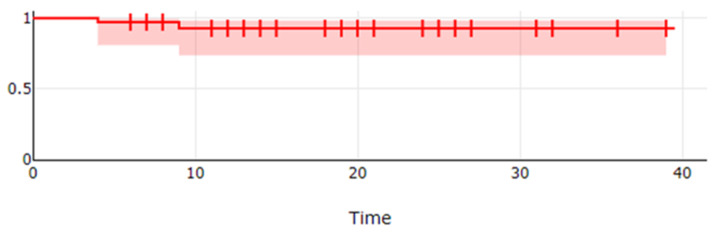
The actuarial 3-year local control of patients affected by POF and treated by IRT.

The actuarial 3-year LC was 94%, as reported in Figure 1.

Three local recurrences were observed, including two marginal (one nasal vestibule and one lip) and one central recurrence (nasal vestibule). No lymph node recurrences were recorded. In all cases of recurrence, salvage surgery was performed.

The median follow-up was 24 months (range between 6 months and 40 months). Acute and late toxicity was regularly assessed during scheduled follow-up (both clinical and radiological) and, noticeably, no G3 or G4 toxicities were registered. Regarding G1/G2 grades, they were present in most patients, with edema and crusts as the most commonly recorded. They resolved spontaneously a few weeks after the end of the treatment.

**Figure 2:**
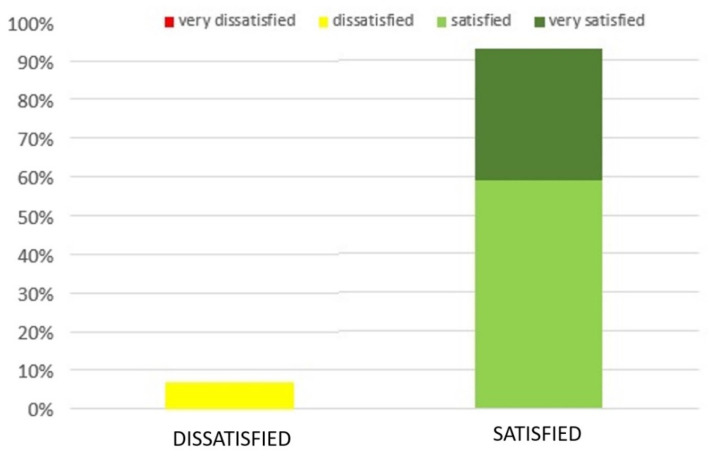
Patients’ satisfaction after treatment with IRT.

Regarding patients’ satisfaction, we followed the aforementioned scale and we found that overall 93% of patients were satisfied; only 7% of the patients were not completely satisfied with the final cosmetic result, as shown in Figure 2.

**Figure 3:**
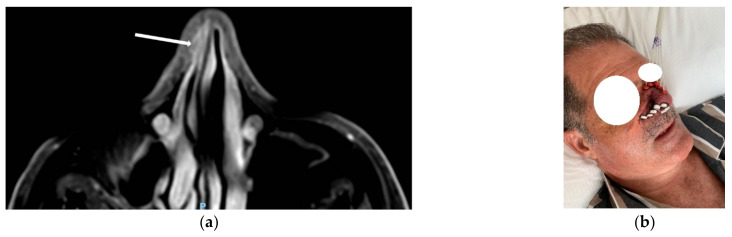

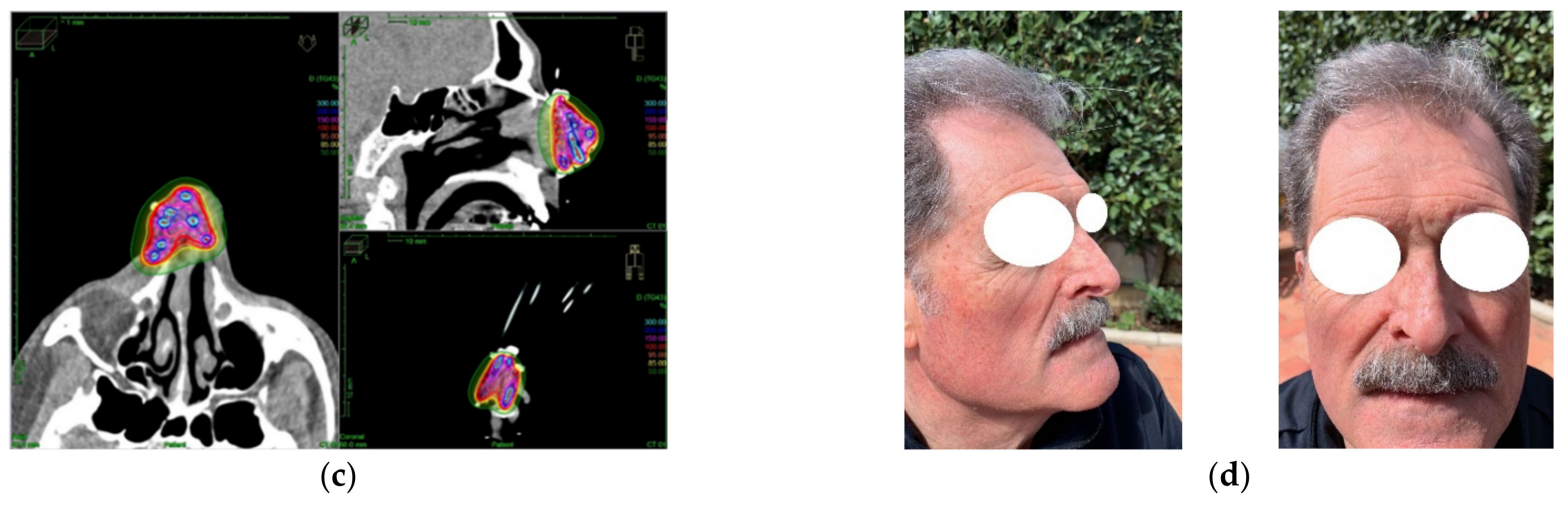
(**a**) MRI with initial diagnosis. (**b**) Implant with plastic tubes and buttons sutured to the skin. (**c**) Treatment plan with isodoses. (**d**) Cosmetic outcome 1 year after IRT. The arrow indicates the primary lesion of the nose vestibule.

An example of a nasal vestibule implant with relative treatment plan and final clinical outcome is reported in Figure 3.

**Figure 4:**
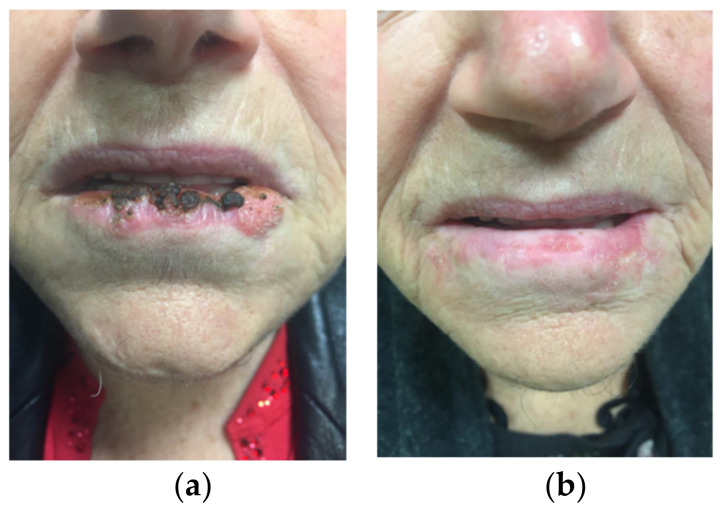
(**a**) Inferior lip cancer before treatment; (**b**) inferior lip cancer 1 year after IRT.

In Figure 4, an example of inferior lip cancer at diagnosis and 1 year after IRT is reported.

On the whole, we reported the results of 40 patients affected by PFC and treated by HDR interstitial IRT with a median follow-up of 24 months and an actual 3-year LC of 94%; noticeably, the rate of patients’ satisfaction with the cosmetic result was 93%.

## 4. Discussion

In this mono-institutional series of patients affected by PFC and treated by IRT, we report a 3-year LC of 94% and a high subjective satisfaction grade.

The results presented in our study are comparable in terms of LC with surgical results from similar series.

In fact, in the large surgical series by de Freitas et al., studying around 102 patients with face cancers in critical sites, the reported disease-free margin rate was 94.1%, which is comparable with the results presented in our series [19].

Regarding the cosmetic outcome and patients’ satisfaction, it is important to note that the overall degree of satisfaction was highly encouraging. There are several points to highlight when considering IRT over surgery in the face region, and the most compelling include the comparable local control rates, optimal cosmetic results and the feasibility in fragile and elderly patients [20].

Notably, a major advantage of IRT over surgery is that, in order to adequately cover the clinical margins (CTV) around the visible tumor (GTV), it is only necessary to add additional catheters, without negatively affecting the cosmetic outcome [17].

Another key element is that IRT may be used also in fragile and elderly patients without additional risks, because it is usually well tolerated.

In our study, we obtained patient satisfaction in 93% of the cases, and only 7% of the patients were not completely satisfied with the final cosmetic result; such results are in line with the data recently presented by Brovchuk et al., who reported, in a large retrospective series of patients treated by a combination of contact HDR radiotherapy and HDR IRT, that excellent cosmetic results were achieved in 79.9%, good cosmetic results in 17.8% and fair results in 1.7% [21].

Regarding existing experiences in the literature about the use of interstitial HDR IRT in the “H zone”, it is necessary to clarify that a few authors have collectively reported about their experiences in the entire face area, considered to be at high risk of recurrence, while data about single subsites, i.e., nasal vestibule, lips and eyelids, are largely available.

In the mid-1970s, there was a strong debate within the oncological community about the possibility of a therapeutic strategy that could provide both an organ and a function sparing approach. In the context of this active debate, with specific regard to the nasal vestibule, the first conservative approaches using radiotherapy in the clinical practice were published in 1976 by Wang, who was the first researcher to show that preserving both organ and function is feasible also in the nasal vestibule cancers [22].

Considering that, today, carcinoma of the nasal vestibule still has no internationally accepted TNM classification, the most relevant contribution provided by Wang was the proposal of a dedicated staging system that grouped patients into T1, T2 and T3 according to the local extent (as reported in Table 4), retaining the TNM classification only for the N and M stages.

Another important point to consider is that the classic TNM staging system does not always account for the therapeutic challenges that may arise in the face due to potentially disfiguring surgical procedures.

For this very reason, the European Association of Dermato-Oncology (EADO) has recently proposed a new staging system for BCCs that groups them into five different categories following the criterion of Difficult-To-Treat (DTT) to account for lesion features such as dimension, multiplicity and presence of critical areas nearby. The major concept of this innovative approach is the link between the staging group proposed and the extent of the surgical approach with the possible disfiguring consequences [23].

The Wang staging system and the possibility of delivering IRT HDR treatment plans by means of CT simulation with 3D reconstruction allow us to achieve clinically excellent results with an exclusive approach, preserving the anatomy and reducing side effects [24].

It is important to underline that more refined staging systems are still needed in order to support clinicians with the best therapeutic strategy to achieve patients’ satisfaction.

In several European countries, the use of IRT has recently gained renewed interest. The largest series available was published by Czerwinski et al., who included 153 patients affected by nasal vestibule cancer and treated by IRT. The authors reported 87% and 89% 3-year local control (LC) and 3-year regional control (RC) rates, respectively, with a 94% 5-year disease-specific survival (DSS) rate. A significant element highlighted by the authors of this series is the degree of patients’ satisfaction with the cosmetic results [25].

Another anatomical site where interstitial IRT finds considerable application as an exclusive approach is represented by the lip, where the use of HDR IRT allows us to achieve local control rates that are clinically comparable to surgery in terms of local disease control [26].

In addition, the IRT approach allows for better quality of life concerning functional and aesthetic aspects in comparison to surgery [27].

As with the nasal vestibule, in the case of the lip, large retrospective series are available about HDR IRT; one of the largest is by Guinot et al., who reported their experience on 104 patients with a median follow-up of 51 months and an LC of 95.2%. The authors underlined that side effects recorded with older IRT techniques, such as soft tissue necrosis, bone necrosis and fair–poor cosmesis, have dramatically declined with the advent of modern equipment [28]. The authors reported also a very high 10-year actual LC rate (94.6%) [29].

Eyelid tumors, and more generally periorbital neoplasms, arise in one of the most radiation-sensitive areas due to the importance of both organ and function (sight) preservation. Only a few series have been published due to the rarity of this clinical presentation, as shown by a recent review on the topic [30].

One the largest and most recent articles is by Cisek et al., who confirmed the effectiveness of the exclusive periorbital interstitial IRT, with excellent results in terms of local control as well as functional and cosmetic outcomes in their series including 28 patients [31].

The main limitations of the present series are the retrospective nature of the study and the low number of included patients. Further prospective and possibly multicenter trials are desirable; however, since IRT is not available in all centers, another valid option could be to retrospectively analyze collected data in a standardized manner across different centers [14].

## 5. Conclusions

Periorificial face cancers are challenging because it is important to balance the local control of the disease with a satisfactory cosmetic outcome. Interstitial HDR IRT could be an effective therapeutic option providing adequate disease control and preventing potentially disfiguring surgical approaches. More numerous and standardized studies are warranted to confirm the available evidence.

## Figures and Tables

**Table 1:** Patients’ characteristics.

Factors	*n* (%)
**Sex**	
Male	24 (60%)
Female	16 (40%)
**Age (mean)**	66.5 years (range 39–94)
**Primary site**	
Nasal vestibule	26 (65%)
Lip	10 (25%)
Eyelid	4 (10%)
**T stage**	
Nasal vestibule *	T1 10 (38%)/T2 15 (58%)/T3 1(4%)
Lip **	T1 7 (70%)/T2 3 (30%)
Eyelid **	T1 4 (100%)
**Initial N**	
N−	38 (95%)
N+	2 (5%)
**Histology**	
SCC	30 (75%)
BCC	10 (25%)
**Follow-up (mean)**	24 months (range 6–40)

* Wang staging system; ** TNM staging system.

**Table 2:** Procedure steps.

**Step 1: Pre-planning**
a. CT or MRI to define the ideal position and insertion of the catheters
b. CT-based pre-planning
c. Catheter insertion calculation with reference to bony landmarks
**Step 2: Implant technique**
a. Bimanual catheter implantation carried out under local or general anaesthesia
b. Catheter insertion carefully avoiding injury
c. Catheters sewn to the skin for stability
**Step 3: Treatment planning and delivery**
a. CT definition of the actual catheter position for 3D treatment planning
b. CT-based IRT planning and optimization
c. Treatment delivery

**Table 3:** Primary sites, doses and fractionation.

Primary Site	Dose	Fractionation
Nose vestibule	44 Gy	3 Gy b.i.d. (first and last fraction 4 Gy)
Lip	45 Gy	5 Gy b.i.d.
Eyelid	49 Gy	3.5 Gy b.i.d.

**Table 4:** Wang’s staging system.

Category	Definition
T1	Limited to the nasal vestibule, relatively superficial and involving 1 or more sites within the nasal vestibule
T2	Extended from the nasal vestibule to the adjacent structures, such as the upper nasal septum, upper lip, philtrum, skin of the nose and/or nasolabial fold, but they are not fixed to the underlying bone
T3	Massive, with extension to the hard palate, bucco-gingival sulcus, large portion of the upper lip, upper septum, turbinate and/or paranasal sinus, fixed with deep muscle or bone involvement

Modified from reference [22].

## Data Availability

Not applicable.
